# The Outcome of Post-Surgical Hip Prosthesis Rehabilitation: Results from a Monocentric Cohort Study

**DOI:** 10.3390/jcm14041276

**Published:** 2025-02-14

**Authors:** Maria Chiara Garifi, Alessandra Cartocci, Giovanni Guarducci, Francesco Praino, Ileana Sanguineti, Simone Cristiani, Anna Maria Gentile, Nicola Nante

**Affiliations:** 1 Post Graduate School of Public Health, University of Siena, 53100 Siena, Italy; giovanni.guarducc@student.unisi.it (G.G.); s.cristiani@student.unisi.it (S.C.); nicola.nante@unisi.it (N.N.); 2 Department of Medical Sciences, Surgical and Neurological Sciences, University of Siena, 53100 Siena, Italy; alessandra.cartocci@dbm.unisi.it; 3 Rehabilitation Unit, Private Hospital San Michele, 17031 Albenga, Italy; frank.praino@gmail.com (F.P.); ileanasanguineti84@gmail.com (I.S.); 4 General Management Unit, Private Hospital San Michele, 17031 Albenga, Italy; annamgentile@gmail.com; 5 Department of Molecular and Developmental Medicine, University of Siena, 53100 Siena, Italy

**Keywords:** outcome, orthopedic rehabilitation, hip prothesis, post-surgical

## Abstract

**Background**: With the increase in life expectancy, more patients are undergoing total hip arthroplasty, primarily due to the rising incidence of osteoarthritis. The outcomes of rehabilitation following these surgical interventions are influenced by various factors. This study aims to explore the impact of age, gender, and body mass index (BMI) on pain and functional and rehabilitative outcomes after surgical hip prosthesis rehabilitation. **Methods**: We enrolled all patients admitted to a private clinic from January 2021 to December 2023 for rehabilitation after unilateral hip arthroplasty. For each patient, we collected data of Barthel Index, Tinetti Scale, Numeric Rating Scale (NRS), and range of motion (ROM) at the beginning and end of the hospitalization. We assessed whether the evaluated outcomes differed based on gender, age, and BMI using the Mann–Whitney and Kruskal–Wallis tests. **Results**: A total of 2.167 patients were studied (56% female and 36.5% over 75 years old). Male patients, adults (18–64 years), and those with a BMI < 30 showed higher values of Barthel Index, Tinetti Scale, and ROM at both admission and discharge (*p* < 0.05), along with significantly lower NRS scores. Each subgroup based on age, gender, and BMI showed an improvement in NRS (difference between admission and discharge) of at least 40%, it was about 50% for men and adults. The improvement in ROM (difference between admission and discharge) was more than 10% in both active and passive flexion, around 20% for passive abduction, and 50% for active abduction, with no significant differences based on age, gender, or BMI. **Conclusions**: Despite the absence of specific contraindications for arthroplasty procedures, a high BMI, age over 75 years, and female gender are associated with slightly worse functional and rehabilitation outcomes compared to other patients undergoing the same procedures. A preoperative screening for the evaluation of osteopenia, osteoporosis, and BMI could be a valuable tool for studying and improving outcomes in these patients.

## 1. Introduction

The steady increase in joint surgeries is linked to the aging population and the growing prevalence of osteoarthritis and other risk factors that necessitate such procedures [1].

According to Italian National Institute of Statistics data, the population is aging, primarily due to the increased life expectancy at birth, which has significantly risen over the last century due to various factors such as the reduction in infant mortality, improved living standards, healthier lifestyles, advances in healthcare, and more [2]. This phenomenon leads to an increasing number of elderly patients who, because of the increased incidence of osteoarthritis in this age group, will have to undergo procedures such as total hip arthroplasty and total knee arthroplasty [3].

Elderly patients today lead healthier lives and seek greater mobility and independence. Positive outcomes of arthroplasty treatment have also been observed in these patients [4,5,6]. In addition to osteoarthritis, inflammatory arthritis, fractures, dysplasia, bone tumors, and other disorders can also necessitate these surgical interventions, involving an increasingly broader and younger population. Although outcomes may vary due to differences in joint structure and underlying medical conditions, most patients experience significant long-term improvement after these procedures [7,8].

Factors influencing the development of osteoarthritis include weight, gender, previous joint injuries, joint dysplasia, and unusual sports activities. Age is the most common factor, as the function of chondrocytes and the ability to synthesize adequate proteoglycan aggregates decrease with age, making cartilage tissue less resistant to cytokines and mechanical stress [9,10]. Additionally, age is an important risk factor for postoperative complications, discharge to a post-acute care facility, and hospital readmission [11,12]. If these operations are performed across different age groups, the average age of the patient is usually between 68 and 74 years [13,14]. Obesity is also a significant factor, as it leads to repeated high axial load stress on joint surfaces, causing cartilage degeneration, irregular development, and impaired healing [15,16]. Orthopedic surgeons are increasingly challenged to assess a patient’s risk during and after surgery, often relying on body mass index (BMI) values. A BMI of 40 kg/m^2^ is often considered a threshold; many surgeons may avoid performing total arthroplasty above this level [17,18]. The impact of gender on preoperative expectations and their fulfillment after total arthroplasty has not been sufficiently investigated. Research indicates that women are more prone to hip osteoarthrosis and typically endure greater functional limitations and pain before undergoing total arthroplasty compared to men [19,20]. Previous studies have suggested that women might experience similar or even greater improvements in function and pain after surgery compared to men. However, they often do not reach the same level of functionality and pain relief as men in the postoperative period [21,22,23,24]. Pain, the main symptom, is primarily assessed through patient-reported outcomes [25,26,27,28,29]. Essentially, assessing risk factors related to increased mortality is crucial for improving perioperative care and patient outcomes [30,31,32]. Physical therapy improves mobility, walking endurance, and overall physical health, as reported by patients, which are central goals of hospital rehabilitation. For patients who have undergone hip surgery [33], or who suffer from multiple sclerosis [34] or Parkinson’s disease [35,36], the effectiveness of exercise is supported by evidence from randomized controlled trials and meta-analyses. Early rehabilitation after knee and hip surgery leads to faster improvement in mobility and reduces overall costs [37,38], in addition to significantly impacting quality of life.

The aim of this study was to evaluate patients’ age, gender, and BMI difference on the functional outcome after unilateral hip arthroplasty through intensive postoperative rehabilitation in hospital.

## 2. Materials and Methods

### 2.1. Study Design and Setting

A monocentric cohort study was conducted. We enrolled a total of 2167 patients. All patients who were consecutively admitted to a specialized clinic (San Michele of Albenga, Savona, Italy) for hospital/intensive rehabilitation between January 2021 and December 2023 were enrolled. A post hoc power analysis was performed to evaluate the power for each objective. The sample size of 2167 was sufficient to have at least 95% power for each hypothesis.

The inclusion criteria for patients were unilateral total hip arthroplasty, ≥18-year-old patients, and consent to data treatment.

### 2.2. Rehabilitation Protocol

The care facility follows an innovative bio-psycho-socio-environmental rehabilitation protocol that highlights the unique characteristics of the structure. Upon the patient’s arrival (mostly on the 3rd–5th day), an individualized rehabilitation plan is established through a document that includes the patient’s personal data, BMI, comorbidities, joint mobility with data from the psychiatric evaluation, and achievable goals. The situation is assessed daily by the multidisciplinary team of the clinic, who assigns personalized therapy as needed. Daily exercises include joint and muscle kinesitherapy (postural exercises, passive mobilization, activities to restore joint mobility, muscle tone, and trophism, assisted mobilization, various exercises for postural control, strengthening of the gluteal and hip muscles, and gait control). As the patient gains independence, they are progressively involved in additional global motor rehabilitation activities (such as active mobilization exercises for the cervical spine, breathing exercises, obstacle courses, etc.). Moreover, when weather conditions allow, the patient can access the equipped park, where they follow a path that includes stairs, inclined planes, obstacles, slalom poles, equipment for upper limb movement, and weight-bearing activities for the lower limbs. Physical activity is complemented by educational sessions aimed at increasing patient awareness and self-management once they return home.

The facility follows a bio-psycho-socio-environmental rehabilitation model, enabling the earliest possible functional recovery by engaging patients in a holistic therapeutic approach for several hours a day. The goal is not only to address physical motor issues but also to provide an educational process that enhances psycho-functional and socio-emotional well-being. Intensive activities take place in the modern physiotherapy center, the panoramic gym, the exclusive “Pine Park”, the multipurpose hall, and, seasonally, the “La Rada del Gabbiano” beach, all scientifically equipped with special attention to the environmental context [39].

### 2.3. Variables

The outcomes considered were the total Barthel Index (range: 0–100), total Tinetti Score (range: 0–28), NRS pain (range 0–10) [40], hemoglobin levels, length of hospital stay, and range of motion (ROM). The ROM outcomes included passive and active flexion (passive flexion 90° and active flexion 85°), passive and active abduction (passive abduction 30° and active abduction 25°), and passive and active extension (0°) [39]. Hemoglobin (Hgb) was considered at target for discharge when ≥10 or stable for three days. The total Barthel Index with a range ≥ 90 and the total Tinetti Score with a range ≥ 24/28 [39]. These variables were analyzed as percentage change (Δ) between admission and discharge and as absolute values at admission and discharge. The analyzed variables that could influence the outcomes were age, sex, and BMI as potential influencing factors. Age was categorized into the following groups: adults (18–64 years), elderly (65–74 years), and very elderly (≥75 years). BMI (body mass index) was classified according to the criteria defined by the World Health Organization as normal weight, overweight, and obese [41]. The “underweight” category was not considered since it was underrepresented.

### 2.4. Statistical Analysis

Descriptive analysis was conducted by estimating absolute frequencies and percentages for qualitative variables, and median and interquartile range (IQR) or mean and standard deviation for quantitative variables. The Mann–Whitney test was performed to assess differences in outcomes based on gender, while the Kruskal–Wallis test was used to evaluate differences in outcomes between three age ranges (18–64, 65–74, and ≥75) and three BMI ranges (18.5–24.9, 25–29.9, and ≥30). All analyses were conducted at a 5% significance level. Analyses were performed using STATA version 16 (StataCorp LLC., College Station, TX, USA).

## 3. Results

Table 1 shows the characteristics of the 2167 patients enrolled. The mean age was 69.6 ± 10.89, and 953 patients (44.0%) were male; 36.5% of patients were very elderly (≥75 years old) and 20% were obese.

In Table 2, the median value of the outcome variables at admission and at discharge are reported. The ROM outcomes increased. The median NRS pain during the day was 4.00 at admission and 2.00 at discharge. The median Barthel score was 80.00 at admission and 90.00 at discharge, while the Tinetti Scale score was 20.00 at admission and 25.00 at discharge.

Table 3 shows the results by gender for patients undergoing unilateral hip arthroplasty at admission (AD) and at discharge (DIS).

The NRS pain scores at admission show that women reported a higher median pain score of 5 compared to 4 for men, which decreased to 3 for women and 2 for men at discharge.

The Barthel Index revealed a median of 80 for men and 75 for women at admission, with a median delta change of 12.5. Both genders had a Tinetti score of 20 at admission; however, by discharge, the median score improved to 26 for men and 25 for women, with men showing a delta change of 23.80 and women 25.

Active flexion increased from a median of 80 for both men and women at admission to 85 at discharge, with a delta change of 12.5. Active abduction started at a median of 15 for both genders, increasing to 25 at discharge, with men showing a 50% improvement and women a 66.66% improvement. The median length of stay was 13 days for men and 14 days for women. Hemoglobin levels at admission were higher in men (11.4 g/dL) compared to women (10.6 g/dL), with slight increases to 11.5 g/dL for men and 10.8 g/dL for women at discharge.

The remaining data (delta NRS, passive flexion at admission, at discharge, and its delta, passive adduction at admission, at discharge, and its delta, and passive and active extension at admission, at discharge, and its delta, as well as delta hemoglobin values) do not show statistical significance (*p* > 0.05). Therefore, gender did not significantly influence these variables.

Table 4 presents result by age group for patients undergoing unilateral hip arthroplasty, highlighting statistically significant differences (*p* < 0.05) that suggest age affects outcomes.

At admission, the median NRS pain score is 4.5 for adults, the elderly, and the super elderly, with the super elderly showing more variability. By discharge, the median pain score decreases to 2 for adults and elderly and to 3 for the super elderly, with a delta change of 50 for adults and 42.86 for the other groups, with increased variability among the super elderly.

Regarding the Barthel Index, adults and the elderly start with a median of 80, while the super elderly start at 75. By discharge, the median increases to 90 for adults and elderly, and to 85 for the super elderly, with delta changes of 12.5 for the younger groups and 13.33 for the super elderly. The Tinetti score at admission is uniformly 20 across all age groups, but by discharge, adults reach a median of 26, elderly 25, and super elderly 24, with the delta change indicating progressively higher improvements for the super elderly.

In terms of range of motion, passive flexion reaches 90° at discharge for all groups. Active flexion improves from 80° for adults and the elderly and 75° for the super elderly at admission to 90° for adults and 85° for the other groups at discharge. Passive abduction at discharge is 30°, with adults showing more variability, while active abduction at discharge is 25°, with variability between adults and the super elderly.

The median length of stay is consistently 4 days across all age groups. Hemoglobin levels at admission show a median of 11.20 for adults, 10.80 for the elderly, and 10.70 for the super elderly. By discharge, the median increases to 11.50 for adults and 11.10 for the elderly, while remaining unchanged for the super elderly, with delta changes of −3.39 for adults, −2.04 for the elderly, and 0 for the super elderly.

Additionally, the data with a *p*-value > 0.05 are also presented, including passive flexion at admission with its delta, the delta of active flexion, passive adduction at admission with its corresponding delta value, active adduction at admission with its corresponding delta, and passive and active extension at admission and discharge. Lastly, the data regarding the delta value of passive and active extension at admission and discharge are missing. Since these data do not have a *p*-value < 0.05, they do not show statistical significance; therefore, age did not influence these variables in our sample.

Table 5 presents the results by BMI category for patients undergoing unilateral hip arthroplasty, highlighting statistically significant differences (*p* < 0.05) that suggest BMI impacts outcomes.

At admission, underweight and normal-weight patients report a median NRS pain score of 4, while overweight and obese patients report a median of 5. By discharge, the median pain score drops to 2 across all groups, except for the obese group, which has a median score of 3.

The Tinetti score at discharge shows a uniform median of 25 across all BMI categories.

In terms of flexion, passive flexion at admission is 85° across all groups, with the obese group showing more variability. By discharge, passive flexion improves to 90° for all groups, with a delta change of 5.88°. Active flexion at admission is 75° for the underweight and obese groups, and 80° for the normal-weight and overweight groups. At discharge, the underweight group reaches 90°, while the other groups reach 85°, with delta changes of 13.33° for the underweight and obese groups and 12.5° for the others.

Regarding abduction, passive abduction at admission is 20° for the underweight group and 25° for the others. By discharge, active abduction is uniformly 25° across all groups. Passive extension at discharge has a median of 0° for all groups, with a delta change of 50° for the obese group and 100° for the normal-weight and overweight groups; this was not recorded for the underweight group. Delta changes for active extension show 200° for the obese group, 100° for the overweight group, 50° for the normal-weight group, and were not recorded for the underweight group.

Lastly, obese patients have the highest median hemoglobin levels (11.1 g/dL) at both admission and discharge, compared to the other BMI categories.

Finally, BMI does not have a significant impact on several variables, including delta NRS, Barthel Index at admission, discharge, and its delta, Tinetti Index at admission and its delta, passive and active adduction at discharge and their deltas, passive extension at admission, active extension at admission and discharge, and length of stay, as the *p*-values did not indicate statistical significance (*p* < 0.05).

## 4. Discussion

The aim of this study was to evaluate the difference in patients’ characteristics (i.e., age, gender, and BMI) in rehabilitation outcomes after surgical hip prosthesis rehabilitation.

The median length of stay was 14 days, without differentiating between hospitalizations of residents in Liguria and from outside the region, which is usually shorter for the latter [42].

We now analyze, in order, the influence of gender, age, and BMI on the studied variables. Given the high number of outcomes considered and the exploratory nature of our analysis, we opted for simpler statistical methods rather than performing multivariate regression. For this reason each factor was independently analyzed.

Several studies showed a higher prevalence of hip or knee osteoarthritis in women, accompanied by greater functional limitations and pain before undergoing total hip or knee arthroplasty (THA/TKA). In our study more than half were women, which is in line with the higher prevalence of hip arthroplasty procedures in female patients [19,20]. Data from previous studies conducted in the same clinic [43,44] confirm that for unilateral hip replacement there were gender differences in the patients’ perception of pain and discomfort. Women have a higher pain and discomfort threshold compared to men.

Gender differences were observed in the values of Barthel Index and Tinetti Scale scores, favoring men; in fact, both scores were higher in men at both admission and discharge. However, at discharge, both sexes showed significant improvements in functional independence. In 2017, the Tinetti Scale also showed higher values in men [44]. In contrast, studies from 2014 [43] indicated greater recovery in the Barthel Index and Tinetti Scale for women. Additionally, our results suggest significant improvements for all the ROM outcomes.

In our study, we then investigated the influence of age on the variables and found some interesting differences.

The analysis of the NRS pain reveals notable differences among adults, elderly, and very elderly patients, both at admission and discharge. Although perceived pain at admission is similar across these groups, there is a significant difference at discharge, with the very elderly reporting slightly higher pain levels compared to the elderly and adults. However, a significant reduction in pain is observed in all three age groups by discharge.

From a physiotherapy point of view, the results suggest that during the hospital stay, both adults and the elderly experienced significant improvements in their ability to perform daily activities, as evidenced by increased Barthel scores at discharge compared to admission. However, discharge scores are lower for the very elderly compared to the other two groups. Between admission and discharge, the very elderly group tend to have a slightly greater change in Barthel scores compared to the other two groups during the observation period. This aligns with the data from a study in 2017 [44], showing greater improvements in active flexion, Barthel, and Tinetti indices in younger patients. Conversely, the 2014 [43] data indicate that older patients showed greater recovery. However, in relative terms, older patients benefitted more from physiotherapy. Our data are consistent with these previous studies. Our findings are in line with the existing literature. Studies by Nilsdotter et al. [45] and Jude et al. [46] have shown that advanced age is a predictor of less favorable functional outcomes after total hip arthroplasty (THA]. Older patients tend to achieve lower post-surgery functional outcomes compared to younger patients. Conversely, Smith et al. [47] reported that younger age is associated with excellent functional outcomes after THA, suggesting that younger patients are more likely to achieve positive functional results compared to older patients. The literature presents a controversy regarding the impact of age on outcomes after THA [45,46,47,48,49,50,51,52] with respect to pain improvement, physical function, and quality of life. This controversy persists due to the limited number of studies, most of which are conducted in single institutions with small sample sizes. Additionally, these studies use varying criteria to define advanced age and employ heterogeneous methods to assess patient outcomes post-THA. In summary, despite efforts by various authors to predict outcomes after THA, the impact of age remains a debated issue that requires further research on a larger scale and with standardized methodologies. Some studies reported good treatment outcomes after total joint arthroplasty even in patients of extreme age [4,5,6]. The lesser improvement in functional mobility with increasing age can be attributed to the inherently higher levels of comorbidities, general frailty, and lower muscle mass in older patients. In our study cohort, we observed that elderly patients often had lower preoperative performance levels, suggesting that surgeons might be less inclined to recommend surgery for the elderly until their clinical condition worsens. Another aspect that warrants attention, considering the aging population, is the decrease in bone mineral density, such as osteopenia or osteoporosis, observed in patients over 75 years old, including post-menopausal women. This could explain the lower rehabilitation recovery rates among these patients. This observation suggests the need for including a screening process in the preoperative evaluation, as these conditions are often undiagnosed or untreated.

Additionally, aging is associated with an increased risk of falls due to age-related changes, including cognitive issues, incontinence, muscle mass and function loss, balance impairment, slower reaction times, and deteriorating vision [53]. Among the elderly, women appear to be at higher risk of fall-related injuries [54].

Finally, we assessed the influence of BMI on the studied variables, which will be presented shortly in order.

The NRS scores indicated significant differences between weight categories at both admission and discharge. Obese participants had higher scores at both time points, suggesting greater symptom severity in this group. Additionally, pain scores during the day were significantly different at admission but not at discharge, indicating symptom homogenization during treatment except for a minimal portion of obese patients. According to the literature, obesity significantly influences osteoarthritis development through biomechanical and physiological mechanisms [55]. Increased BMI elevates joint cartilage loading forces, contributing to tissue damage [56]. Adipose tissue releases adipokines, proteins causing inflammation and cartilage degradation, increasing osteoarthritis risk and the need for total joint arthroplasty [57]. Individuals with a BMI over 40 are 8.5 times more likely to undergo total hip arthroplasty compared to non-obese individuals [58]. Other studies associate obesity with poorer long-term functional outcomes after hip or knee surgery [59,60], including reduced mobility [60] and inadequate physical activity levels [61]. Postoperative complication rates related to obesity are inconsistent in the literature, with some studies finding no significant associations [62,63,64] and others noting increased complication rates with higher BMI [65,66,67,68,69,70]. Our study found that overweight and obese patients reported slightly lower preoperative and postoperative functional and health status scores. Pain intensity and interference were worse preoperatively for obese patients; they improved postoperatively but remained higher than in normal- and underweight patients. This highlights the importance of preoperative BMI evaluation and potentially advising weight loss before surgery. However, denying arthroplasty solely to motivate weight loss may not be effective, and considering surgical risks, a balanced approach is needed.

## 5. Conclusions

The results of this study align with those obtained from cases treated at the same facility during previous time periods. However, an approximate two-day increase in the average length of stay was observed to achieve these results. This can be attributed to clusters of COVID-19 infections that were not always diagnosed due to the mildness of symptoms, often limited to a fever that nonetheless slows daily rehabilitation activities. Additionally, the precautionary observation of incoming patients, imposed by the hospital management to shield the facility from sporadic outbreaks in the referring surgical departments, contributed to this increase. Our research highlights that the lesser improvement in functional mobility with advancing age can be attributed to various factors, including comorbidities, general frailty, and lower muscle mass in elderly patients. Additionally, the presence of conditions such as osteopenia or osteoporosis in older individuals can negatively impact postoperative rehabilitative recovery, suggesting the need for preoperative screening for these undiagnosed or untreated conditions. The study underscores the significant impact of BMI on surgical outcomes. Obese patients present with higher pain scores preoperatively and report less improvement postoperatively compared to their normal-weight counterparts. Despite similar functional recovery across BMI categories at discharge, the greater severity of preoperative symptoms and slower recovery in obese patients emphasize the need for preoperative weight management. The increased risk associated with a high BMI further supports the importance of incorporating weight reduction strategies into preoperative care to optimize surgical outcomes and recovery. This approach should be carefully evaluated, as simply denying arthroplasty may not effectively motivate weight loss and could have negative implications for the patient’s overall health.

However, further studies are needed to fully understand the impact of weight loss on joint arthroplasty outcomes and whether such an approach could improve overall results for obese and overweight patients. Preoperative screening for osteopenia, osteoporosis, and BMI assessment could be a valuable tool in preventing contraindications and improving outcomes in these patients, as well as in appropriately prioritizing these interventions.

It would be interesting to study the outcomes of patients who undergo arthroplasty and are evaluated by their general practitioner and specialists regarding BMI and the possible presence of osteopenia/osteoporosis. These patients would likely experience a different, less physically and emotionally demanding rehabilitation process, leading to a quicker and higher-quality social reintegration.

## Figures and Tables

**Table 1:** Description of the studied sample (2167 patients).

Variables		N (%)/Median [IQR]
Gender	Males	953 (44.0%)
	Females	1214 (56.0%)
Age	Adults (18–64)	658 (30.4%)
	Elderly (65–74)	718 (33.1%)
	Very elderly (≥75)	791 (36.5%)
BMI	Underweight	30 (1.4%)
	Normal weight	764 (35.3%)
	Overweight	909 (41.9%)
	Obese	434 (20.0%)
Length of stay		14.00 [13.00–15.00]
Days from surgery to admission		4.00 [3.00–5.00]

**Table 2:** Description of clinical variables.

Variables	Median [IQR]		
	Admission	Discharge	Delta
Hgb	10.90 [10.00–1.80]	11.10 [10.20–12.00]	1.80 [2.63–7.14]
Passive flexion	85.00 [80.00–90.00]	90.00 [90.00–90.00]	5.88 [0–12.5]
Active flexion	80.00 [70.00–80.00]	85.00 [85.00–90.00]	12.5 [6.25–21.42]
Passive abduction	25 [20.00–25.00]	30.00 [30.00–30.00]	20 [20–50]
Active abduction	15.00 [10.00–20.00]	25.00 [25.00–25.00]	50 [25–100]
Passive extension	0.00 [0.00–0.00]	0.00 [0.00–0.00]	100 [0–100]
Active extension	0.00 [0.00–0.00]	0.00 [0.00–0.00]	100 [0–100]
NRS	5.00 [3.00–6.00]	2.00 [2.00–3.00]	50 [20–62.5]
Barthel Index	80.00 [75.00–80.00]	90.00 [85.00–90.00]	12.5 [12.5–20]
Tinetti Scale	20.00 [19.00–21.00]	25.00 [24.00–26.00]	23.80 [18.18–30]

**Table 3:** Gender difference in variables studied.

Variables	Gender		*p*-Value
	Males (953)	Females (1214)	
NRS AD	4 [3–6]	5 [3–7]	<0.001
NRS DIS	2 [1–3]	3 [2–4]	<0.001
Delta NRS	50 [20–62.5]	42.86 [20–60]	0.119
Barthel Index AD	80 [75–80]	75 [75–80]	<0.001
Barthel Index DIS	90 [90–90]	90 [85–90]	<0.001
Delta Barthel	12.5 [12.5–20]	12.5 [12.5–20]	<0.001
Tinetti total AD	20 [19–21]	20 [19–21]	<0.001
Tinetti total DIS	26 [24–26]	25 [23–26]	<0.001
Delta Tinetti	23.80 [18.18–30]	25 [18.18–33.33]	<0.001
Passive flexion AD	85 [80–90]	85 [80–90]	0.197
Passive flexion DIS	90 [90–90]	90 [90–90]	0.504
Delta passive flexion	5.88 [0–6.25]	5.88 [0–12]	0.877
Active flexion AD	80 [70–80]	75 [70–80]	<0.001
Active flexion DIS	85 [85–90]	85 [85–90]	<0.001
Delta active flexion	12.5 [5.88–20]	12.5 [6.25–21.43]	0.005
Passive abduction AD	25 [20–25]	25 [20–25]	0.066
Passive abduction DIS	30 [30–30]	30 [30–30]	0.079
Delta passive abduction	20 [20–50]	20 [20–50]	0.1899
Active abduction AD	15 [10–20]	15 [10–20]	<0.001
Active abduction DIS	25 [25–25]	25 [25–25]	0.001
Delta active abduction	50 [25–100]	66.66 [25–133.33]	0.016
Passive extension AD	0 [0–0]	0 [0–0]	0.485
Passive extension DIS	0 [0–0]	0 [0–0]	0.766
Delta passive extension	100 [100–100]	50 [100–25]	0.560
Active extension AD	0 [0–0]	0 [0–0]	0.135
Active extension DIS	0 [0–0]	0 [0–0]	0.731
Delta active extension	100 [100–100]	50 [100–0]	0.259
Length of stay	13 [12–15]	14 [13–15]	<0.001
Hgb AD	11.4 [10.2–12.5]	10.6 [9.8–11.5]	<0.001
Hgb DIS	11.5 [10.6–12.5]	10.8 [10.1–11.6]	<0.001
Delta Hgb	−1.65 [−7.05–2.88]	−1.88 [−7.22–2.59]	0.497

**Table 4:** Age difference in the studied variables.

Variables	Age			*p*-Value
	Adults (658)	Elderly (718)	Very Elderly (791)	
NRS AD	4.5 [3–6]	4.5 [3–6]	4.5 [3–7]	0.037
NRS DIS	2 [2–3]	2 [2–3]	3 [2–3]	<0.001
Delta NRS	50 [25–66.6]	42.86 [16.67–60]	42.86 [20–60]	0.038
Barthel AD	80 [75–80]	80 [75–80]	75 [65–8]	<0.001
Barthel DIS	90 [90–90]	90 [90–90]	85 [85–9]	<0.001
Delta Barthel	12.5 [12.5–18.7]	12.5 [12.5–20]	13.33 [12.5–23]	<0.001
Tinetti AD	20 [20–22]	20 [19–21]	20 [17–20]	<0.001
Tinetti DIS	26 [25–26]	25 [24–26]	24 [22–26]	<0.001
Delta Tinetti	23.81 [18.18–30]	25 [18.18–30]	25 [18.18–36.8]	<0.001
Passive flexion AD	85 [80–90]	85 [85–90]	85 [80–90]	0.249
Passive flexion DIS	90 [90–90]	90 [90–90]	90 [90–90]	<0.001
Delta passive flexion	5.882 [0–6.25]	5.88 [0–12.5]	5.88 [0–12.5]	0.500
Active flexion AD	80 [70–80]	80 [70–80]	75 [70–80]	0.015
Active flexion DIS	90 [85–90]	85 [85–90]	85 [85–90]	<0.001
Delta active flexion	12.5 [6.25–20]	12.5 [6.07–21.43]	12.5 [6.25–21.43]	0.623
Passive abduction AD	25 [20–25]	25 [20–25]	25 [20–25]	0.056
Passive abduction DIS	30 [30–35]	30 [30–30]	30 [30–30]	<0.001
Delta passive abduction	20 [20–50]	20 [20–50]	20 [20–50]	0.307
Active abduction AD	15 [10–20]	15 [10–20]	15 [10–20]	0.056
Active abduction DIS	25 [25–30]	25 [25–25]	25 [20–25]	<0.001
Delta active abduction	50 [25–100]	50 [25–100]	50 [25–100]	0.371
Passive extension AD	0 [0–0]	0 [0–0]	0 [0–0]	0.232
Passive extension DIS	0 [0–0]	0 [0–0]	0 [0–0]	0.884
Delta passive extension	-	-	-	-
Active extension AD	0 [0–0]	0 [0–0]	0 [0–0]	0.256
Active extension DIS	0 [0–0]	0 [0–0]	0 [0–0]	0.416
Delta active extension	-	-	-	-
Length of stay	4 [3–5]	4 [3–5]	4 [3–5]	<0.001
Hgb AD	11.2 [10.2–12.3]	10.8 [9.9–11.9]	10.7 [9.9–11.6]	<0.001
Hgb DIS	11.5 [10.76–12.4]	11.1 [10.3–12.1]	10.7 [10–11.6]	<0.001
Delta Hgb	−3.39 [−8.49–1.72]	−2.04 [−7.12–1.86]	0 [−5.44–4.45]	<0.001

**Table 5:** BMI difference in the studied variables.

Variables	BMI				*p*-Value
	Underweight (30)	Normal Weight (764)	Overweight (909)	Obese (434)	
NRS AD	4 [3–5]	4 [3–6]	5 [3–6]	5 [3–7]	0.002
NRS DIS	2 [1–3]	2 [2–3]	2 [2–3]	3 [2–4]	<0.001
Delta NRS	50 [18.25–58.33]	43.65 [20–60]	50 [20–62.5]	42.85 [20–60]	0.674
Barthel AD	75 [70–80]	80 [75–80]	80 [75–80]	80 [75–80]	0.075
Barthel DIS	90 [85–90]	90 [85–90]	90 [85–90]	90 [85–90]	0.190
Delta Barthel	13.33 [12.5–20.0]	12.5 [12.5–20]	12.5 [12.5–20]	12.5 [12.5–20]	0.570
Tinetti AD	20 [18–20]	20 [19–21]	20 [19–21]	20 [19–21]	0.457
Tinetti DIS	25 [21–26]	25 [24–26]	25 [24–26]	25 [24–26]	0.028
Delta Tinetti	25 [15–35.24]	25 [18.18–30]	23.809 [18.18–30]	23.8 [15–30]	0.325
Passive flexion AD	85 [80–90]	85 [80–90]	85 [80–90]	85 [80–85]	0.004
Passive flexion DIS	90 [90–90]	90 [90–90]	90 [90–90]	90 [90–90]	<0.001
Delta passive flexion	5.882 [0–12.5]	5.882 [0–6.25]	5.882 [0–12.5]	5.882 [0–12.5]	0.03
Active flexion AD	75 [70–80]	80 [70–80]	80 [70–80]	75 [70–80]	<0.001
Active flexion DIS	90 [85–90]	85 [85–90]	85 [85–90]	85 [85–90]	<0.001
Delta active flexion	13.33 [12.5–28.57]	12.5 [6.066–2]	12.5 [6.25–21.42]	13.33 [6.25–20]	0.007
Passive abduction AD	20 [20–25]	25 [20–25]	25 [20–25]	25 [20–25]	0.038
Passive abduction DIS	30 [30–30]	30 [30–30]	30 [30–30]	30 [30–30]	0.118
Delta passive abduction	40 [20–50]	20 [20–50]	20 [20–50]	20 [20–50]	0.13
Active abduction AD	15 [10–15]	15 [10–20]	15 [10–20]	15 [10–20]	0.116
Active abduction DIS	25 [25–25]	25 [25–25]	25 [25–25]	25 [25–25]	0.012
Delta active abduction	66.66 [50–10]	50 [25–100]	50 [25–100]	50 [25–100]	0.513
Passive extension AD	0 [0–0]	0 [0–0]	0 [0–0]	0 [0–0]	0.798
Passive extension DIS	0 [0–0]	0 [0–0]	0 [0–0]	0 [0–0]	0.036
Delta passive extension	–	100 [100–100]	100 [100–100]	50 [0–100]	0.022
Active extension AD	0 [0–0]	0 [0–0]	0 [0–0]	0 [0–0]	0.68
Active extension DIS	0 [0–0]	0 [0–0]	0 [0–0]	0 [0–0]	0.227
Delta active extension	–	50 [0–100]	100 [100–100]	200 [0–200]	0.003
Length of stay	14.5 [13–15]	14 [13–15]	13 [12–15]	14 [13–15]	0.175
Hgb AD	10.45 [9.7–11.4]	10.7 [9.9–11.6]	11 [10–12]	11.1 [10.2–12.1]	<0.001
Hgb DIS	10.7 [9.8–11.4]	10.9 [10.1–11.7]	11.2 [10.4–12.1]	11.25 [10.4–12.2]	<0.001

## Data Availability

The data that support the findings of this study are available on request from the corresponding author. The data are not publicly available due to privacy or ethical restrictions.
